# Near-infrared dye marking for thoracoscopic resection of small-sized pulmonary nodules: comparison of percutaneous and bronchoscopic injection techniques

**DOI:** 10.1186/s13019-018-0697-6

**Published:** 2018-01-12

**Authors:** Takashi Anayama, Kentaro Hirohashi, Ryohei Miyazaki, Hironobu Okada, Nobutaka Kawamoto, Marino Yamamoto, Takayuki Sato, Kazumasa Orihashi

**Affiliations:** 1 Division of Thoracic Surgery, Department of Surgery II, Kochi Medical School, Kochi University, Kohasu Oko Nankoku Kochi, 783-8505 Japan; 2 Department of Circulation Control, Kochi Medical School, Kochi University, Kohasu Oko Nankoku Kochi, 783-8505 Japan

**Keywords:** Indocyanine green fluorescence, Near-infrared spectroscopy, Small-sized pulmonary nodules, Video-assisted thoracoscopic surgery

## Abstract

**Background:**

Minimally invasive video-assisted thoracoscopic surgery for small-sized pulmonary nodules is challenging, and image-guided preoperative localisation is required. Near-infrared indocyanine green fluorescence is capable of deep tissue penetration and can be distinguished regardless of the background colour of the lung; thus, indocyanine green has great potential for use as a near-infrared fluorescent marker in video-assisted thoracoscopic surgery.

**Methods:**

Thirty-seven patients with small-sized pulmonary nodules, who were scheduled to undergo video-assisted thoracoscopic wedge resection, were enrolled in this study. A mixture of diluted indocyanine green and iopamidol was injected into the lung parenchyma as a marker, using either computed tomography-guided percutaneous or bronchoscopic injection techniques. Indications and limitations of the percutaneous and bronchoscopic injection techniques for marking nodules with indocyanine green fluorescence were examined and compared.

**Results:**

In the computed tomography-guided percutaneous injection group (*n* = 15), indocyanine green fluorescence was detected in 15/15 (100%) patients by near-infrared thoracoscopy. A small pneumothorax occurred in 3/15 (20.0%) patients, and subsequent marking was unsuccessful after a pneumothorax occurred. In the bronchoscopic injection group (*n* = 22), indocyanine green fluorescence was detected in 21/22 (95.5%) patients. In 6 patients who underwent injection marking at 2 different lesion sites, 5/6 (83.3%) markers were successfully detected.

**Conclusion:**

Either computed tomography-guided percutaneous or bronchoscopic injection techniques can be used to mark pulmonary nodules with indocyanine green fluorescence. Indocyanine green is a safe and easily detectable fluorescent marker for video-assisted thoracoscopic surgery. Furthermore, the bronchoscopic injection approach enables surgeons to mark multiple lesion areas with less risk of causing a pneumothorax.

**Trial Registration:**

UMIN-CTR R000027833 accepted by ICMJE. Registered 5 January 2013.

## Background

Lung cancer is the leading cause of death worldwide. Computed tomography (CT) is currently the most effective screening method for detecting lung cancer and reducing lung cancer mortality [1, 2]. Bronchoscopy and percutaneous needle biopsy are performed for pulmonary lesions with a strong suspicion of malignancy based on CT findings. However, in cases where lung lesions are small in size, present in the periphery of the lung, or close to the visceral pleura, thoracoscopic biopsy may be performed [3]. Therefore, there is an increasing need for wedge resection of small-sized pulmonary nodules by means of video-assisted thoracic surgery (VATS) for both the diagnosis and treatment of lung cancer.

Nevertheless, localisation of small-sized pulmonary nodules is challenging. In particular, ground-glass nodule (GGN) lesions do not alter the surface of the visceral pleura, and the elevation of tumours cannot be perceived in the deflated lung during VATS; thus, GGNs are difficult to localise. Small-sized pulmonary nodules are often marked prior to VATS by using a VATS marker such as a hook-thread [4], spiral wire needle [5], microcoil [6], fiducial marker [7], or colour dyes such as methylene blue [8]; each of these is injected into the lung near the target using a CT-guided percutaneous injection approach. Alternatively, barium [9] or lipiodol [10] can be injected with CT guidance, and the labelled nodules can be intraoperatively detected by fluoroscopy. Radiotracer-guided localisation can also be used with gamma-emitting radioisotopes (technetium 99, Tc99m) for localising nodules with CT-guided injection [11, 12]. Gamma-ray emission signals can be detected intraoperatively using a gamma probe.

Bronchoscopic markers are an alternative to VATS markers. With a bronchoscopic approach, multiple markers can be placed in the lung without injuring the visceral pleura or causing a pneumothorax. Various materials, such as methylene blue [13], indigo carmine [14], and fiducial markers [15], as well as methods such as radiofrequency identification (RFID) have been used [16]. Navigation bronchoscopy technology, such as virtual bronchoscopy and electromagnetic navigation bronchoscopy (ENB) [17, 18], are used to guide the tip of the bronchoscope to the target lesion area. We have previously reported the concept of ENB-guided bronchoscopic injection of indocyanine green (ICG) and localisation of infrared ICG-fluorescence (ICG-FL) to localise small-sized pulmonary nodules using a porcine model [19]. In that study, we determined the optimal dose, concentration, and volume of ICG to produce a small ICG-FL spot in the lung parenchyma that could be visualised using a near-infrared (NIR) thoracoscope.

In the current study, we translated this basic research into a clinical investigation of the feasibility and the efficacy of ICG-FL marking to localise small-sized pulmonary nodules in human patients. We compared CT-guided percutaneous marking and bronchoscopic marking to clarify the benefits and detectability of these two techniques.

## Methods

### Patient enrolment

Thirty-seven patients who were scheduled for VATS wedge resection of a non-solid, partly solid, or solid pulmonary nodule with a maximum nodule diameter < 20 mm located in the peripheral part of the lung, were included in this study. The study period was from January 2013 to December 2014 for CT-guided percutaneous marking, and from January 2015 to December 2016 for bronchoscopic marking.

### Ethics, consent, and permissions

Patients provided written informed consent to participate in the study and for individual patient data to be published. This study was approved by the Institutional Review Board of Kochi Medical School, Kochi University.

### VATS marking procedures

An ICG/iopamidol mixture was prepared by diluting ICG 100-fold (2.5 mg/ml, 10 ml; Daiichi-Sankyo, Tokyo, Japan) with iopamidol (Lopamiron 370, Bayer, Leverkusen, Germany). A 1 ml syringe was filled with the ICG/iopamidol mixture and connected to either a CT-guided percutaneous needle or a bronchoscopic needle, and the needle lumen was filled with the marking solution prior to the marking procedure.

**Fig. 1:**
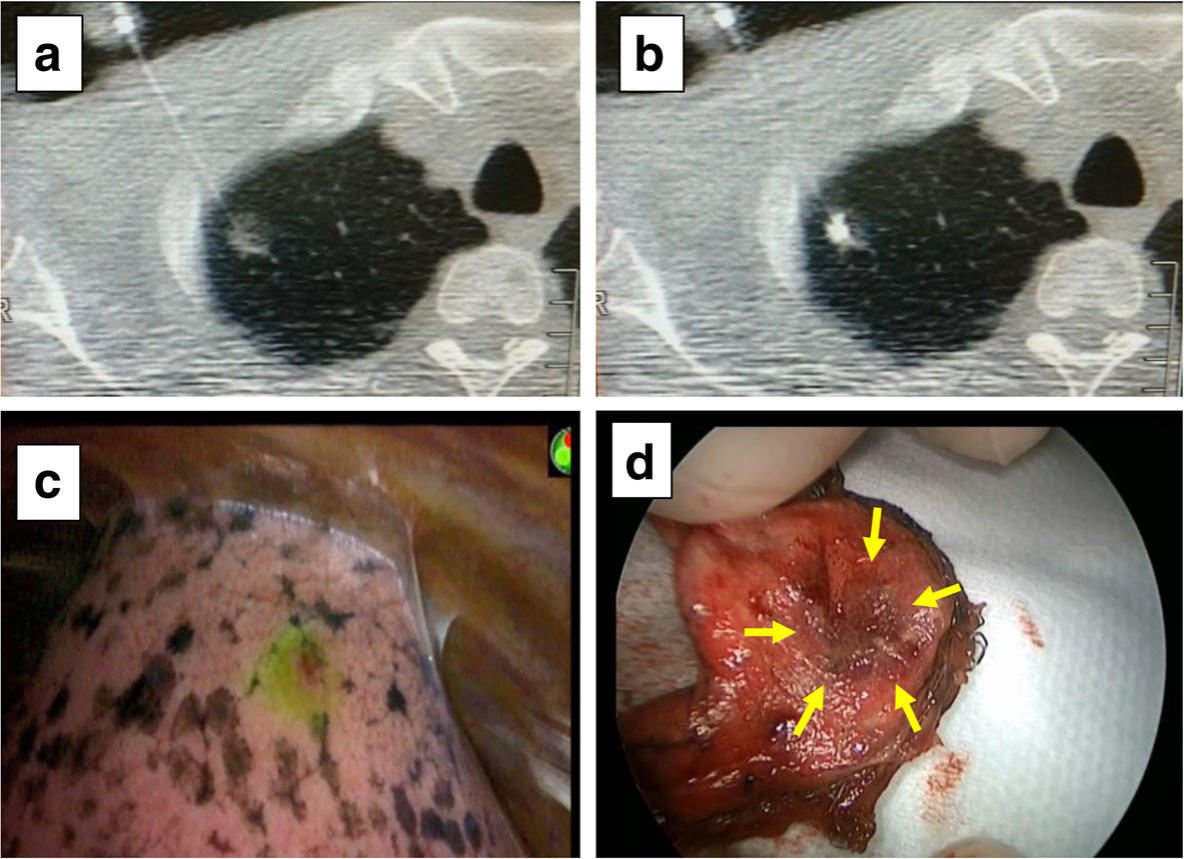
Computed tomography (CT)-guided percutaneous marking, near-infrared (NIR) fluorescence detection, and wedge resection. **a**: A 23-gauge needle was inserted into the lung near a ground-glass nodule under CT guidance. **b**: A mixture (0.1 ml) of 100-fold diluted indocyanine green and iopamidol was injected into the lung parenchyma. **c**: The indocyanine green fluorescence was detected using NIR thoracoscopy (PINPOINT^®^, Novadaq). **d**: A resected lung specimen from the surgical field. The target pulmonary nodule is indicated by yellow arrows

The CT-guided percutaneous marking procedure was performed as follows (Fig. 1). The patient was placed in either the prone, supine, or lateral position, depending on the location of the lesion. After a preliminary scan, the CT scanner was focused on the area of the pulmonary nodule. After providing local anaesthesia with lidocaine, a 23-gauge needle filled with marking solution was injected near the pulmonary nodule. Fifty microlitres of ICG/iopamidol marking solution was injected under real-time imaging using CT fluoroscopy. CT scans were performed repeatedly for 30 min after the injection to monitor for the occurrence of pneumothorax or air embolism. The total duration of the procedure was within 90 min, including observation time. In consideration of the possibility of late-onset pneumothorax, the marking procedure was performed in the morning, and surgery was performed on the same afternoon.

**Fig. 2:**
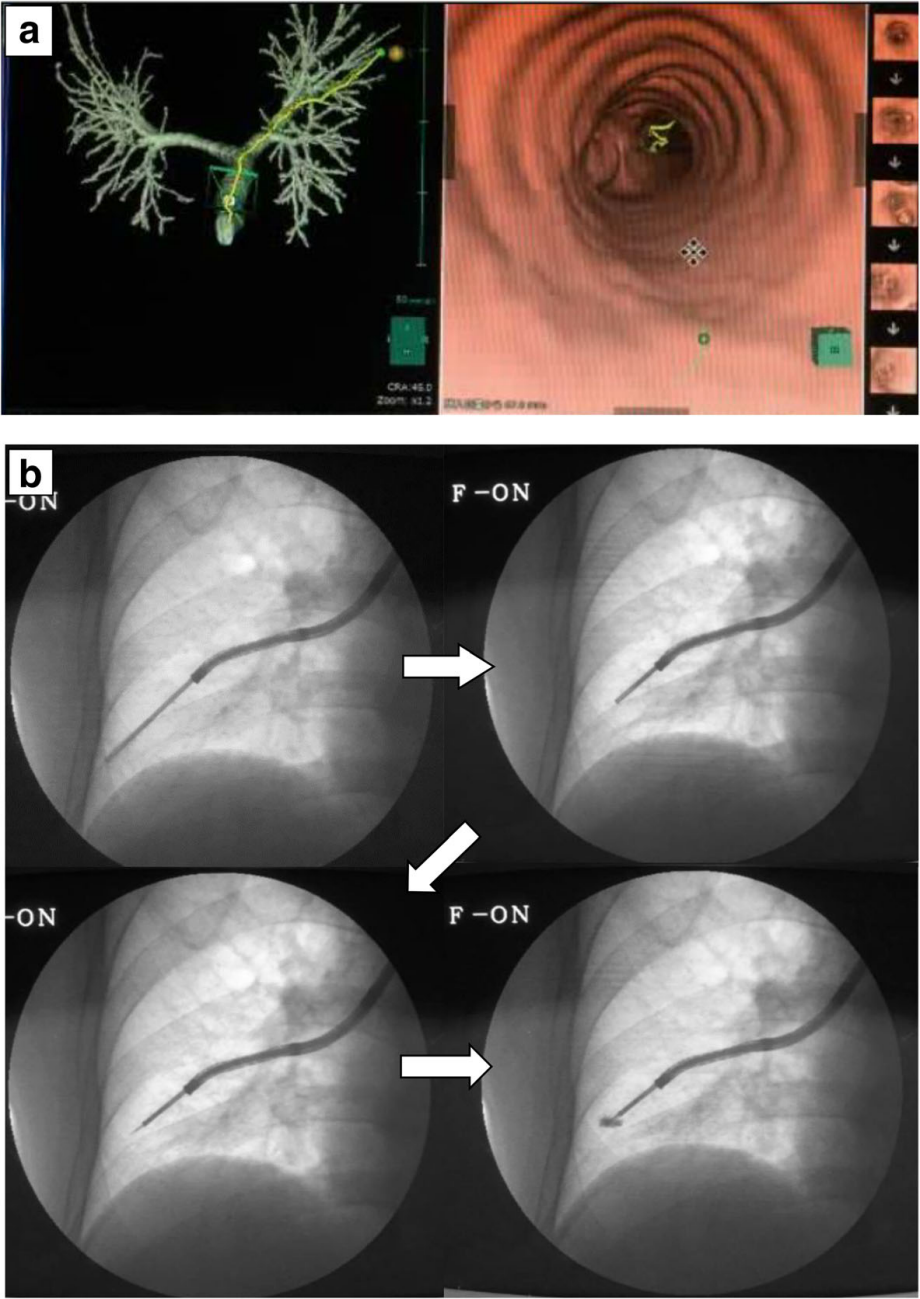
Virtual bronchoscopy-guided bronchoscopic marking. **a**: Virtual bronchoscopy image created by Synapse Vincent (Fuji Film, Tokyo, Japan) to visualize the best path to reach the target pulmonary nodule. **b**: A thin bronchoscope was inserted into the peripheral bronchus based on the virtual bronchoscopy images. A transbronchial aspiration cytology (TBAC) needle enclosed within an outer sheath was advanced to the peripheral end of the bronchus until the operator could feel resistance. The outer sheath was retracted 3 cm, and 1 cm of the TBAC needle tip was exposed from the outer sheath. The TBAC needle and the outer sheath were advanced 1 cm to penetrate the lung parenchyma through the bronchial wall. The indocyanine green/iopamidol marking dye was injected through the TBAC needle into the lung parenchyma

The bronchoscopic marking procedure was performed as follows (Fig. 2). Virtual bronchoscopy navigation images were generated from the CT DICOM data using the Synapse Vincent (Fuji Film, Tokyo, Japan) volume analyser. Bronchoscopy was performed according to conventional procedures. The upper airway (nasal passage, oropharynx, vocal cords, and trachea) was topically anesthetised using aerosolised 2% lidocaine and 1% lidocaine gel. After topical anaesthesia, moderate sedation was induced by administrating 2−3 mg of midazolam intravenously. A thin, flexible bronchoscope (P290, Olympus, Tokyo, Japan) was inserted nasally into the tracheobronchial tree. While viewing the virtual bronchoscopy navigation images, the tip of the bronchoscope was guided to the peripheral bronchus near the target lung tumour. A sheath containing a transbronchial aspiration cytology (TBAC) needle was inserted through the accessory channel of the bronchoscope to the peripheral end of the bronchus immediately below the visceral pleura under X-ray fluoroscopy. The sheath was retracted 3 cm from that position, the TBAC needle was exposed 1 cm from the sheath, and then the sheath and needle were advanced together 1 cm to puncture the lung parenchyma. Fifty microliters of ICG/iopamidol marking solution was injected into the lung parenchyma under X-ray fluoroscopy. The duration of bronchoscopic marking procedures was no more than 30 min. A chest CT image was then acquired and the marking point was confirmed. The surgical procedure was performed one day after the bronchoscopic marking.

### Thoracoscopic NIR fluorescence detection and video-assisted thoracoscopic wedge resection

**Fig. 3:**
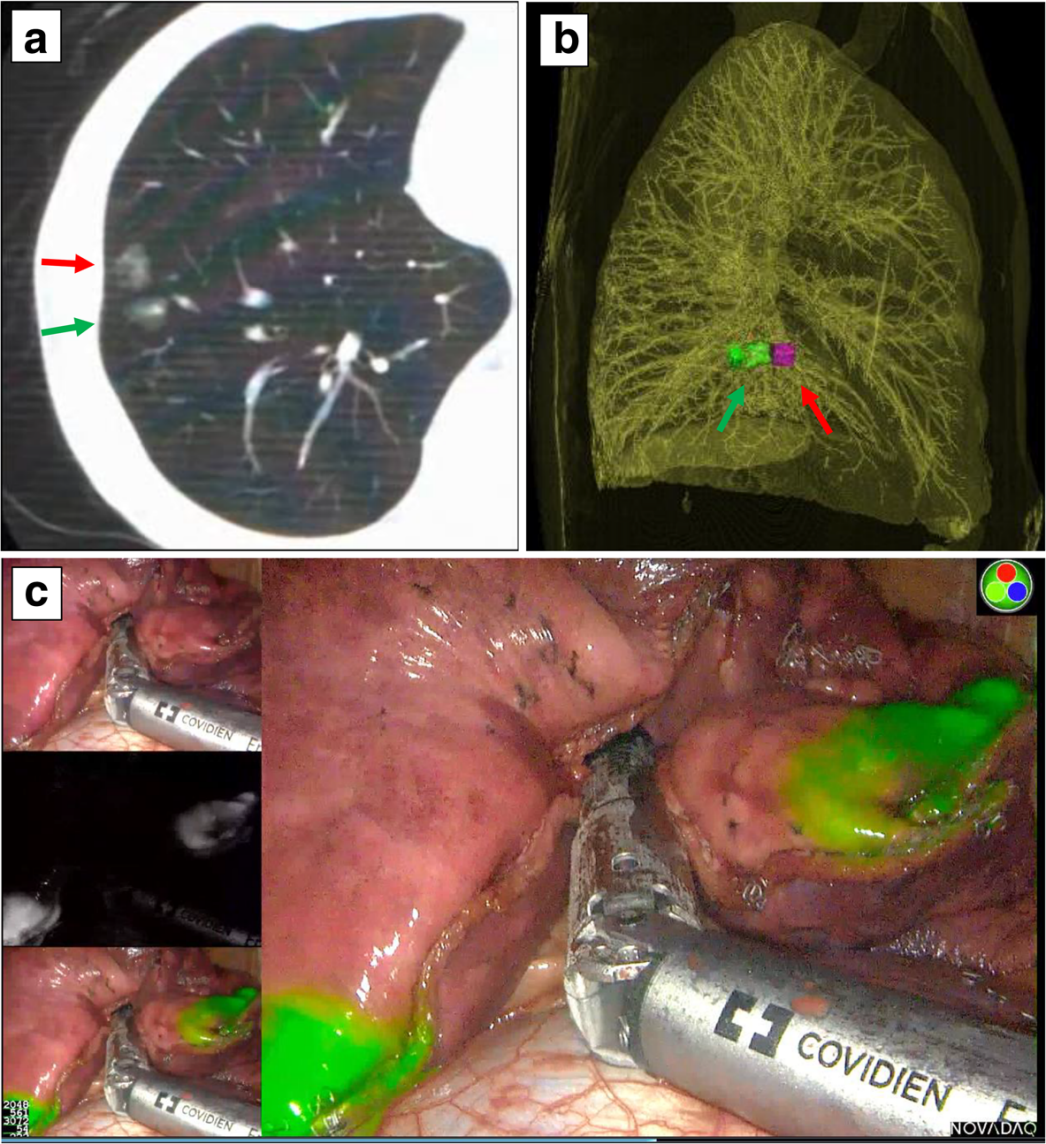
Near-infrared fluorescence marked VATS wedge resection. **a**: computed tomography (CT) scan was performed after a bronchoscopic marking procedure to confirm the marked position. The indocyanine green (ICG)/iopamidol marking dye (green arrow) was injected into a 3 cm dorsal point of the target nodule (red arrow) in the same axial slice of the CT image. **b**: A three-dimensional CT image was constructed to assess the position of both the target pulmonary nodule (red arrow) and the ICG/iopamidol marker (green arrow) in the right anterior basal segment. **c**: During the surgery, ICG fluorescence was detected by the PINPOINT^®^ (Novadaq) endoscopic fluorescence imaging system, and the pulmonary nodule was excised by cutting between the ICG-fluorescence marker and the anterior edge of the basal segment. PINPOINT^®^ visualises white light images without infrared (on the left top), infrared signal only (on the left middle), and a hybrid mode with both the infrared signal and the white light image together (on the bottom left and right)

Two- and three-dimensional CT images of the marking point and the pulmonary nodule were prepared prior to surgery in order to clarify the positional relationship between the marker and the tumour (Fig. 3a, b). During thoracoscopic surgery, ICG-FL was visualised using the PINPOINT^®^ endoscopic fluorescence imaging system (Novadaq, Mississauga, Canada) (Fig. 3c). The part of the lung believed to contain the tumour was partially excised using an automatic suturing device. Successful removal was confirmed using rapid pathological diagnosis.

### Statistical analysis

Any significant differences among the categorized groups were compared using either the two-sided chi-squared test or Fisher’s exact test, and 0.05 was the threshold for statistical significance. All analyses were performed using SPSS version 17.0 (SPSS Inc., Chicago, IL, USA) for Windows (Microsoft Corporation, Redmond, WA, USA).

## Results

In total, 37 patients participated in this clinical trial. Percutaneous marking was performed in 15 patients and bronchoscopic marking in 22 patients. There was no difference in the clinical background data between the two patient groups (Table 1). Sub-centimetre nodules were found in 4 patients in the CT-guided percutaneous needle injection group, and in 13 patients in the bronchoscopic injection group. All pulmonary nodules were located at the 1/3 peripheral side of the lung. Each group included one patient with two pulmonary nodules in the ipsilateral side of lung.

**Table 1:** Patient characteristics

		CT-guided percutaneous needle injection group	Bronchoscopic injection group	*p* Value
Study period		Jan 2013 - Dec 2014	Jan 2015 - Dec 2016	
Patients (n)		15	22	
Age, years		61.5 ± 12.6	64.4 ± 10.0	N.S.
Sex	Male	10	13	N.S.
	Female	5	9	
Tumour	size (mm) size range (mm)	10 ± 3.4 (4.0–17.1)	9.2 ± 3.6 (3.5–16.0)	N.S.
	GGN (n) Solid (n)	12 4	10 13	
	Depth from visceral pleura	9.9 ± 7.7	9.8 ± 8.1	N.S.
	Localization			
	Right superior lobe (S1, S2, S3)	7 (1, 4, 2)	6 (4, 1, 1)	
	Right middle lobe (S4, S5) Right inferior lobe (S6, S7, S8, S9, S10) Left superior lobe (S1 + 2, S3, S4, S5) Left inferior lobe (S6, S8, S9, S10)	1 (0, 1) 4 (0, 0, 1, 2, 1) 3 (2, 1, 0, 0) 1 (0, 0, 1, 0)	1 (1, 0) 8 (0, 1, 2, 3, 2) 4 (3, 0, 1, 0) 4 (0, 1, 2, 1)	

Data presented as median ± standard deviation; GGN: ground-glass nodule; Jan: January; Dec: December; S1: apical segment; S2: posterior segment; S3: anterior segment; right S4: lateral segment; right S5: medial segment; S6: superior segment; right S7: medial-basal segment; right S8: anterior-basal segment; S9: lateral segment; S10: posterior-basal segment; S1 + 2: apico-posterior segment; left S4: superior lingular segment; left S5: inferior lingular segment; left S8: anteriomedial basal segment; N.S.: not significant

**Fig. 4:**
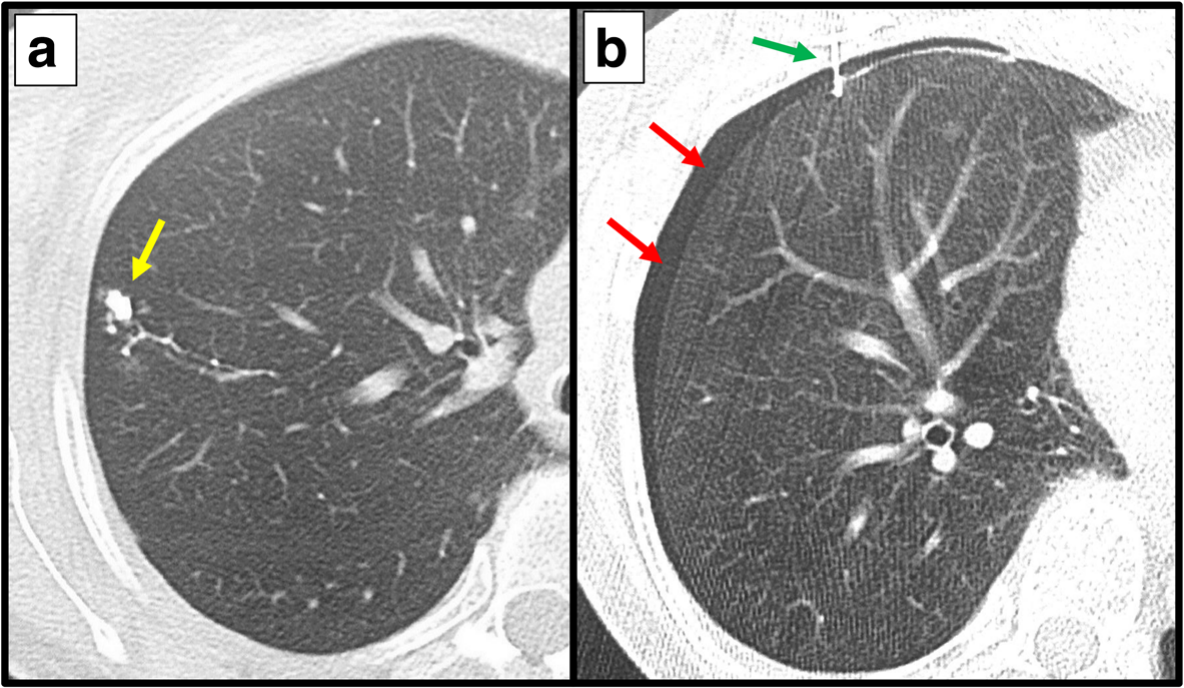
Limitations of the percutaneous needle dye injection approach. **a**: indocyanine green/iopamidol marking dye was injected percutaneously (yellow arrow). **b**: A pneumothorax (red arrow) developed after the first injection, after which the lung was not stably fixed, even when breathing was stopped during the second attempt at percutaneous injection (green arrow)

In the CT-guided ICG-FL marking group, ICG fluorescence was detected using NIR thoracoscopy in all 15 (100%) patients, who all successfully underwent VATS wedge resection. A small pneumothorax occurred in 3/15 (20.0%) patients, none of whom required chest tube drainage. For one patient, a second marking procedure was attempted but was unsuccessful because the pneumothorax disrupted the lung anatomy and the localisation of the lesion (Fig. 4).

**Fig. 5:**
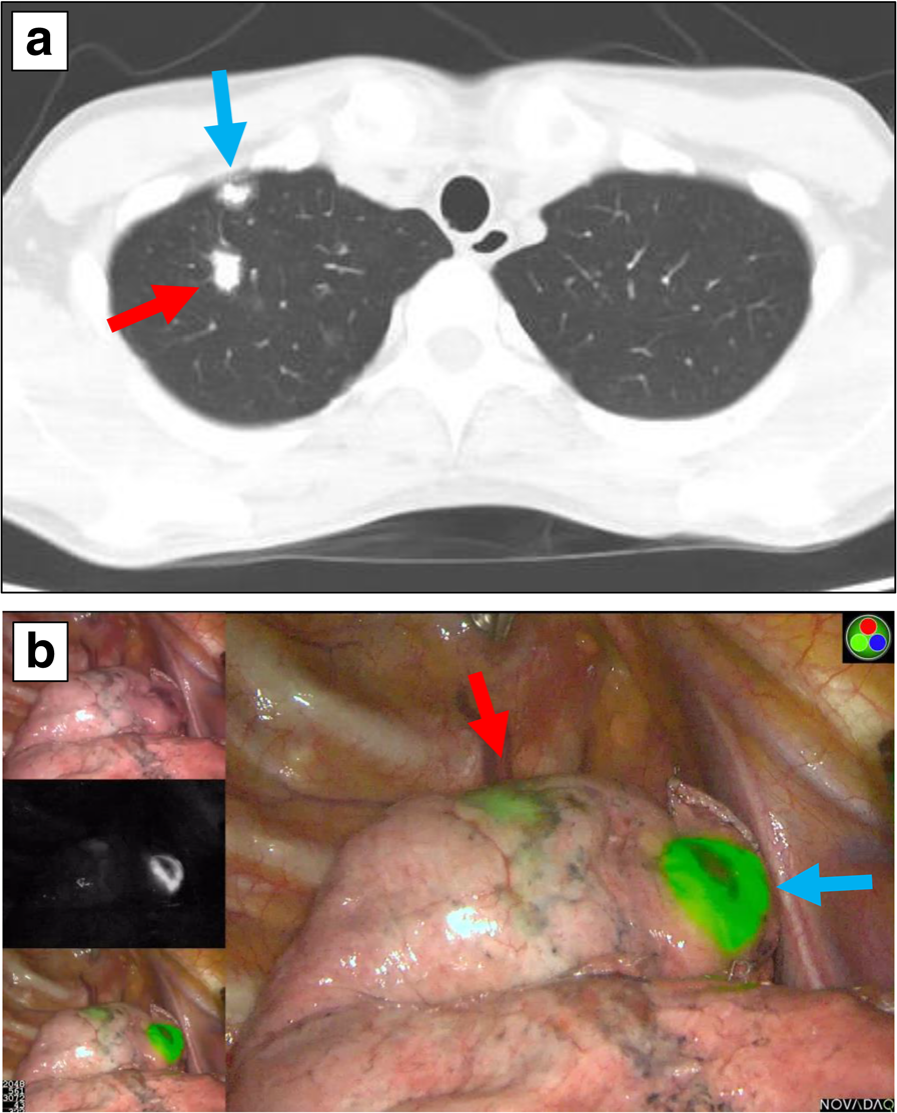
CT (**a**) and near-infrared thoracoscopic (**b**) findings of a case with multiple bronchoscopic indocyanine green fluorescence markings. Two indocyanine green fluorescence markers were injected in both the median (red arrow) and anterior part (blue arrow) of the right apical segment. Both markers were visualised using a near-infrared thoracoscope

Bronchoscopic ICG-FL marking was successful in 20/22 (90.9%) patients (Table 2). In the 16 patients in whom an ICG-FL marker was placed, ICG-FL was detected by NIR thoracoscopy in 15 (93.8%) patients. In the patient in whom the ICG-FL marker could not be detected by NIR thoracoscopy, the ICG-FL marker was injected at a depth of 28 mm from the visceral pleura. In the 6 patients in whom the ICG-FL marker was placed at two different positions in the lung, both markers were detected in 5 (83.3%) patients (Fig. 5). One marker, which was injected at a depth of 30 mm from the visceral pleura, was not detected in a patient who had two markers placed.

**Table 2:** The success rates and complications of VATS marking

No. of markers	CT-guided percutaneous needle injection	Bronchoscopic injection
No. of patients	15	22
Single marking	14/14 (100%)	15/16 (86.0%)
Double marking	0/1 (0%)	5/6 (83.3%)
Complication		
Pneumothorax	3/15 (20%)	0/22 (0%)
Other	0 (0%)	0 (0%)

*VATS* video-assisted thoracic surgery

For the 2 patients in whom the ICG marker was not detectable, the access portal was extended by approximately 3 cm and the surgeon was able to localise the pulmonary nodules by palpation. Ultimately, the target pulmonary nodules were successfully resected with a negative surgical margin in all patients.

Other than a small pneumothorax in 3 patients, no complications were observed throughout the study period.

## Discussion

Marking of a pulmonary mass using ICG-FL was confirmed to be a safe and reliable procedure using both percutaneous and bronchoscopic injection techniques. One of the advantages of NIR fluorescence marking is that NIR fluorescence can be detected using spectroscopy, regardless of the background colour of the lung; this can be especially valuable in patients with a smoking history whose lungs have black deposits. Another advantage of NIR fluorescence is its good tissue transparency properties [20–22]. As we previously demonstrated in an animal study, NIR dye is not necessarily present on the surface of the lung, but the NIR fluorescence of a marker injected into the lung at a depth of 20 mm can be detected by NIR thoracoscopy [19]. In the current study, we injected a small amount of NIR marker (50 μl) into an area close to the tumour based on imaging studies and showed that it could be detected using NIR fluorescence thoracoscopy. Because the volume of ICG/iopamidol marker was as small as 50 μl, and it was injected near the tumour rather than within the tumour, the risk for spreading tumour cells was minimized.

Both percutaneous marking and bronchoscopic marking have benefits. Since percutaneous marking is not dependent on bronchial branching, it can easily be performed by interventional radiologists, using the same technique as CT-guided biopsy. The success rate of a single marking using the CT-guided percutaneous approach was 15/15 (100%). However, CT-guided percutaneous injection can injure the visceral pleura, potentially causing a pneumothorax. In the current study, the authors used a small-sized (23-gauge) needle to minimise damage to the visceral pleura. In our study, only 3/15 (20%) patients who underwent CT-guided percutaneous ICG-FL marking developed a pneumothorax, none of whom required chest tube drainage. However, these small pneumothoraces prevented repeated VATS marking procedures. Thus, the CT-guided percutaneous approach is not suitable for marking multiple lung regions.

Bronchoscopic marking enables the injection of ICG-FL markers into multiple lung sections without injuring the visceral pleura. In addition, the bronchoscopic approach can access regions of the lung that are difficult to reach using a percutaneous approach, such as the mediastinal side and the craniodorsal part that is obscured by the scapula. By referring to guidelines on lung biopsy, the bronchoscopic approach is chosen in preference to the percutaneous approach when accessible by bronchoscopy [23].

The success rate of the bronchoscopic approach was 20/22 (91.0%), which was lower than that of the CT-guided percutaneous approach. Furthermore, the accuracy of virtual bronchoscopy navigation combined with X-ray fluoroscopy guidance was not as accurate as CT-guidance. In fact, we experienced two failed marking procedures. One case was a GGN located in the right apical segment, to which the ICG-iopamidol marker was injected into an area of lung deeper than 20 mm from the visceral pleura. In this case, only posterior-anterior projection X-ray fluoroscopy was used for image-guidance. Lateral projection X-ray fluoroscopy may have increased bronchoscopic marking accuracy. The other case involved GGNs located in the right medial basal segment. Because the segment overlapped with the cardiac shadow, it was technically difficult to inject ICG-iopamidol marker at the correct point. In such a case, CT fluoroscopy is useful for confirming the position of the bronchoscopic needle. Additionally, electromagnetic navigation bronchoscopy may be more accurate than virtual bronchoscopy navigation, and should be evaluated in further studies.

In terms of cost, the CT-guided percutaneous approach involves only the expense of using the CT scanner unless complications, such as severe pneumothorax, occur. On the other hand, the bronchoscopic approach involves the costs of both the bronchoscopy and the CT imaging after the marking procedure.

When the target pulmonary nodule was not located near the bronchial tree, the VATS marker was injected in the vicinity of the pulmonary nodule. It was easy to determine the location of the nodule from the positional relationship between the marker and the tumour. Unlike metal markers, liquid markers do not necessarily have to be removed with the pulmonary nodules because they are absorbed by the body. The currently available devices, such as PINPOINT^®^ and D-light P, have almost equivalent capabilities for detecting ICG NIR fluorescence, and either of these devices can be used for the method described in this study [19].

The use of ICG-FL as a thoracoscopic marker does have some disadvantages. In cases of severe pulmonary emphysema, there is concern that liquid markers do not form a distinct spot but rather diffuse into pulmonary cysts; therefore, ICG may not be ideal in such cases. Moreover, the equipment required to thoracoscopically visualise ICG-FL is expensive and is available only in a limited number of institutions.

## Conclusions

ICG-FL is a VATS marker with good detectability and is capable of deep tissue penetration; ICG-FL can also be distinguished regardless of the tissue background colour. Pulmonary nodules can be marked with ICG-FL using either a CT-guided percutaneous or a bronchoscopic injection technique. Using the bronchoscopic approach, multiple VATS markers can be placed without causing a pneumothorax. ICG-FL using the bronchoscopic injection technique can be especially useful for marking multiple small-sized pulmonary nodules for minimally invasive resection.

## Abbreviations

CT: Computed tomography
ENB: Electromagnetic navigation bronchoscope
GGN: Ground-glass nodule
ICG: Indocyanine green
ICG-FL: Indocyanine green fluorescence
NIR: Near-infrared
TBAC: Transbronchial aspiration cytology
VATS: Video-assisted thoracic surgery
